# Crosslinked Gel Polymer Electrolyte from Trimethylolpropane Triglycidyl Ether by In Situ Polymerization for Lithium-Ion Batteries

**DOI:** 10.3390/gels10010040

**Published:** 2024-01-02

**Authors:** Lei Jin, Hyunmin Lim, Wansu Bae, Subeen Song, Kijong Joo, Hohyoun Jang, Whangi Kim

**Affiliations:** Department of Applied Chemistry, Konkuk University, 268 Chungwon-daero, Chungju-si 27478, Republic of Korea; jinlei8761@naver.com (L.J.); tree3367@naver.com (H.L.); gelp621@naver.com (W.B.); hubsugar@naver.com (S.S.); joo10140@gmail.com (K.J.); 200417450@kku.ac.kr (H.J.)

**Keywords:** gel polymer electrolytes, tri-epoxide monomer, LiFSI, in situ cationic polymerization, lithium-ion batteries

## Abstract

Electrolytes play a critical role in battery performance. They are associated with an increased risk of safety issues. The main challenge faced by many researchers is how to balance the physical and electrical properties of electrolytes. Gel polymer electrolytes (GPEs) have received increasing attention due to their satisfactory properties of ionic conductivity, mechanical stability, and safety. Herein, we develop a gel network polymer electrolyte (GNPE) to address the challenge mentioned earlier. This GNPE was formed by tri-epoxide monomer and bis(fluorosulfonyl)imide lithium salt (LiFSI) via an in situ cationic polymerization under mild thermal conditions. The obtained GNPE exhibited a relatively high ionic conductivity (σ) of 2.63 × 10^−4^ S cm^−1^, lithium transference number (*t*_Li_^+^, 0.58) at room temperature (RT), and intimate electrode compatibility with LiFePO_4_ and graphite. The LiFePO_4_/GNPE/graphite battery also showed a promising cyclic performance at RT, e.g., a suitable discharge specific capacity of 127 mAh g^−1^ and a high Coulombic efficiency (>97%) after 100 cycles at 0.2 C. Moreover, electrolyte films showed good mechanical stability and formed the SEI layer on the graphite anode. This study provides a facile method for preparing epoxy-based electrolytes for high-performance lithium-ion batteries (LIBs).

## 1. Introduction

LIBs were initially commercialized in the 1990s. Since then, their application has been expanded to many things ranging from portable devices to electric vehicles [1,2,3,4]. LIBs present considerable energy density and efficiency while being lightweight and small [5,6,7,8,9]. However, the leakage of electrolytes is always a hot issue in that it can lead to burning and even explosions [10,11,12]. Electrolytes are among the most critical components of LIBs. They play a vital role in lithium-ion transport, battery span life, and cell performance [13,14]. It is well-known that liquid electrolytes are used in mostly LIBs because of their excellent electrochemical performance, especially regarding RT ionic conductivity. Liquid organic solvents can provide a high dielectric constant value, fluidity, and electrode compatibility [15,16,17]. On the other hand, they can lead to reduced thermal stability, low flame retardancy, and liquid leakage of electrolytes [18,19]. Safety concerns of LIBs have promoted the increased development of polymer electrolytes that exhibit a superior thermal stability to liquid electrolytes and excellent mechanical strength to relieve hazards from lithium dendrites. Furthermore, they offer a high energy density to high-capacity LIBs [20,21,22]. Unfortunately, a low σ at RT and considerable interfacial resistance between electrolytes and electrodes limit the practical application of LIBs [23,24]. Modifications of polymer electrolytes have been extensively studied for several decades to address these limitations [25,26]. Typically, an all-solid polymer electrolyte (ASPE) can form an intimate connection with electrodes through high temperature and pressure, resulting in similar ionic conductive values to liquid electrolytes. However, the demanding electrolyte plate preparation process also limits its production scale and application [27,28,29]. On the other hand, many previous works have demonstrated that gel polymer electrolytes can reflect relatively high RT σ and satisfactory electrode interface compatibility, although they have more moderate production procedures than those of all-solid electrolytes [30,31]. The preparation of the GPEs can be divided into physical and chemical methods. Generally, in the physical methods, polymer matrices are dissolved in the solvents and then mixed with additives. The dried polymer film, which is obtained after evaporating solvents, is soaked in the liquid electrolyte solution to form a gel polymer electrolyte. However, the polymer matrices are facial to dissolve or deform into the liquid electrolyte after temperature increases and prolonged aging, resulting in solution leakage and battery performance degradation. In addition, the lower thermal stability of the prepared GPEs limits this method for practical application [32]. In terms of chemical methods, initiators and lithium salts are first dissolved in a monomer or liquid electrolyte to form a precursor electrolyte solution. The polymerization is then carried out by thermal-, radical-, or electrochemical-initiated methods for obtaining the crosslinked GPEs. The chemical methods offer an essay procedure to prepare electrolyte and cell assembly and are also named “in-situ” polymerization. The obtained GPEs provide a high thermal and mechanical stability, promising contact with the electrodes, and satisfactory electrochemical performance [33,34].

In terms of polymer matrices, linear polymers bearing ethylene oxide structures have been broadly investigated due to a high donor number for lithium ions and chain flexibility. Poly(ethylene oxide) (PEO), the most well-known polymer matrix, possesses promising physicochemical properties, especially regarding a high lithium salt solubility and dielectric constant [35,36]. Unfortunately, the high crystal phase in PEO restricts ion mobility, leading to insufficient room temperature σ and difficulty meeting the requirements of electrolytes [37]. Recently, heterocyclic ring monomers have been researched to form polymer electrolytes via ring-opening polymerization to overcome this shortcoming [33,38]. Herein, epoxide monomers are expected to be alternative structure electrolytes owing to their low *T*_g_ and crystallinity, which are essential for polymer chain mobility and ion transport [39]. Moreover, as thermosetting monomers, epoxide monomers exhibit excellent thermal stability to withstand high temperatures. They can maintain good mechanical strength after curing polymerization, which is beneficial for batteries far from lithium dendrites damage [12,40,41,42]. On the other hand, epoxide monomers carrying various functional groups can create considerable variants and easily modify characteristics of polymer electrolytes. Moreover, the ring-opening of epoxy will yield a CH_2_-CH_2_-O- structure, which tends to have a good ion transport ability and high conductive values [43]. Coincidentally, epoxide monomers can offer excellent solubility to lithium salts and are catalyzed by lithium salts, leading to ring-opening polymerization under certain conditions. In brief, lithium salts can react with a trace of moisture and produce Brønsted acid, which continuously attacks the carbon-oxygen bond of epoxy and yields a polymer chain growth [44,45,46]. This reaction process finally results in a crosslinked network polymer electrolyte that is free of impurities. 

On the other hand, trimethylolpropane (TMP) has been increasingly researched in gel polymer electrolytes. The chemical structures of trimethylolpropane offer excellent physical support for the electrolytes. Dong Zhou et al. [47] reported crosslinked trimethylolpropane trimethylacrylate-based gel polymer electrolytes showing high ionic conductivity (>10^−3^ S cm^−1^ at 25 °C) and a wide electrochemical window (>5.0 V). The three-dimensional framework offered excellent electrochemical properties and high mechanical strength. Qiu-Jun Wang et al. [48] prepared an electrolyte presenting improved ionic conductivity and low interfacial resistance due to the TMPTMA providing structure support for liquid parts in the GPEs by chemical crosslinking. 

In the present work, we used trimethylolpropane triglycidyl ether, which bears tri-epoxy functional groups, as a crosslinker to form a polymer matrix. In terms of lithium salts, LiFSI was applied in this study because it had a better ionic conductivity, viscosity, and thermal stability than others. In addition, LiFSI can also offer a good capacity for Li-ion batteries [49,50,51]. Moreover, small amounts of EC/DEC solution can be added into electrolytes due to the EC/DEC well-known dielectric constant and ability to increase [52,53,54]. Gel polymer electrolytes have been successfully prepared by in situ thermal polymerization. The obtained electrolytes exhibited an outstanding battery performance with bright development prospects for epoxy-based gel polymer electrolytes. 

## 2. Results and Discussion

### 2.1. In Situ Polymerization and Properties of GNPEs

**Scheme 1:**
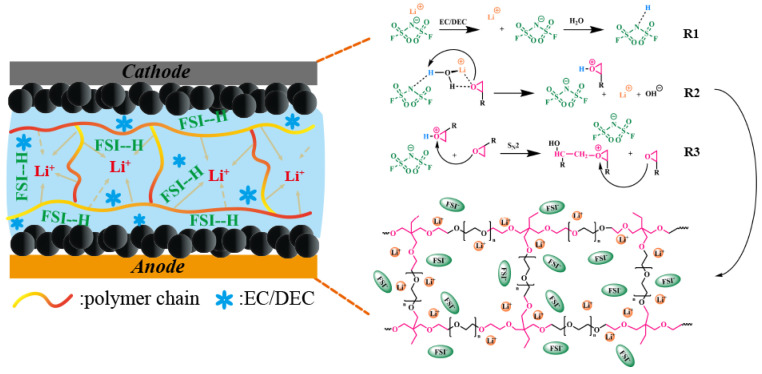
Illustration of in situ cationic ring-opening polymerization catalyzed by LiFSI and the eventual chemical structure of a gel network polymer electrolyte based on TMPTE.

GNPEs were fabricated via in situ cationic ring-opening polymerization of TMPTE using LiFSI as an initiator as described in Scheme 1 and Figure S1 of the Supplementary Materials (SM). Li-ions and sulfonimide anion formed after LiFSI dissociates in EC/DEC could react with protogenic components such as impurity traces of water or alcohols to yield a superacid initiator, H^+^FSIOH^–^ (R1). The Brønsted superacid H^+^FSIOH^–^ then rapidly protonated the cyclic epoxy monomer to form the secondary oxonium species (R2). The cationic ring-opening polymerization was produced under S_N_2 attack by the nucleophilic cyclic ether monomer to secondary oxonium species (R3). Finally, a network polymer was accomplished after continual repetitive attack by cyclic epoxy on the heterocyclic intermediate [55,56]. 

**Figure 1:**
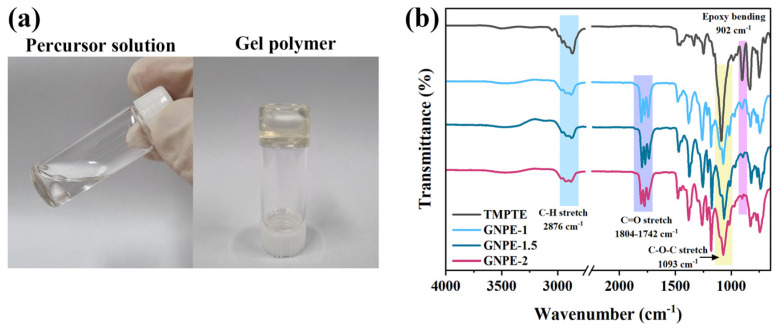
(**a**) Macroscopical comparison of electrolyte state before and after cationic ring-opening polymerization, (**b**) FT IR results of GNPEs compared with pure TMPTE.

Figure 1a macroscopically compares the polymer electrolyte state before and after cationic ring-opening polymerization. All materials, including LiFSI, TMPET, and EC/DEC solvent, can dissolve with each other sufficiently, resulting in a transparent, clean, and viscous precursor solution. The viscosity is increased when the LiFSI mole ratio is increased. After thermal cationic polymerization, an elastic gel polymer electrolyte is formed without excess fluid liquid in the obtained electrolyte, which is beneficial for future coin cell assembly (in Figure 1a right). The ring-opening polymerization was monitored by FT IR. The resulting spectra are shown in Figure 1b. Pure TMPTE presents three fingerprint peaks at 2876, 1093, and 902 cm^−1^ corresponding to C−H, C−O−C stretching, and epoxide bending vibration, respectively. As the reaction proceeded, the intensity of the characteristic peak for epoxide bending declined dramatically and the C=O stretching peak appeared at 1804−1742 cm^−1^ due to the addition of the EC/DEC solvent [57]. These results proved the occurrence of the cationic ring-opening polymerization of TMPTE with LiFSI. Noticeably, the liquid epoxy monomer did not transfer to the all-solid state even after a long time of polymerization, which was inevitable in the gel polymer electrolyte forming process [26]. Figure S2 reflects the polymerization of GNPE-1 at different time points, with a small epoxide bending peak remaining at 902 cm^−1^ even after 7 days.

**Figure 2:**
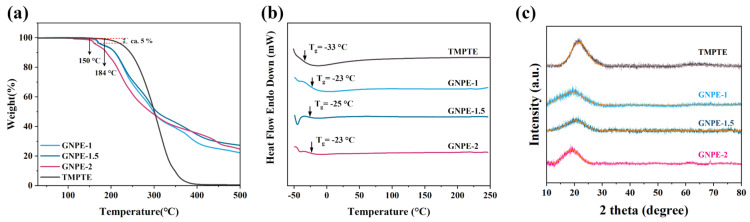
(**a**) TGA, (**b**) DSC result curves, and (**c**) XRD pattern of GNPEs and pure TMPTE.

TGA and DSC were utilized to investigate the thermal properties of the prepared GNPEs. Figure 2a illustrates the epoxy monomer TMPTE with a one-step weight degradation from 184 °C. Because of the addition of lithium salt and EC/DEC solvent, GNPEs presented a ca. 5% weight loss from 150 °C. The secondary weight decomposition of GNPEs was observed above 170 °C for GNPE-1 and GNPE-1.5 and 160 °C for GNPE-2. DSC measurement was carried out at a temperature range of −60 to 150 °C to identify the glass transition temperature of GNPEs. The results are shown in Figure 2b. *T*_g_ values were observed at −23, −25, and −23 °C for GNPE-1, GNPE-1.5, and GNPE-2, respectively. The DSC result revealed that these GNPEs could offer sufficient chain flexibility at RT or sub-zero. Furthermore, there was no exothermic peak in the investigated temperature range for these GNPEs, hinting that these obtained electrolytes presented amorphous phases. Combining TGA and DSC data, the prepared GNPEs were thermally stable from around −25 to 150 °C, values which were close to some PEO and PMMA-based polymer electrolytes. On the other hand, lithium-ion transfer in the polymer matrix could also be conducted over a wide temperature range. 

In addition, XRD analysis was performed to further investigate the crystallization of TMPTE and GNPEs. As shown in Figure 2c, intensely shaped characteristic diffraction peaks were not observed, indicating crystalline phases for TMPTE and GNPEs. Broad diffraction peaks were found at 18~20° for samples, indicating an amorphous phase of both TMPTE and GNPEs, consistent with the DSC results. Moreover, GNPE-2 exhibited a similar theta degree to other polymers but appeared to have a lower theta degree than other samples. It also showed a small peak at 69°, attributed to the fact that GNPE-2 was fabricated with high concentrations of lithium salts, which affect the arrangement of polymers. The lack of impurity peaks in the spectrum also confirmed the purity of the as-prepared electrolytes. These results suggest that the obtained GNPEs can process a facial channel for lithium ions hopping in polymer electrolytes. 

### 2.2. Electrochemical Performances of GNPEs

**Figure 3:**
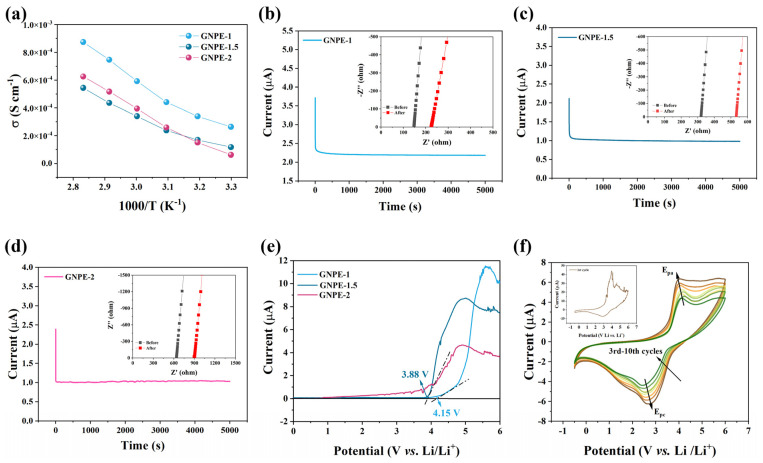
(**a**) Temperature dependent ionic conductivity of GNPEs, (**b**–**d**) chronoamperometry curves of the symmetrical cell Li/GNPEs/Li at a polarization voltage of 10 mV and Nyquist plots of the cell before and after polarization, (**e**) linear sweep voltammetry for the SS/GNPEs/Li foil between 0 and 6 V at a rate of 1 mV s^−1^, (**f**) cyclic voltammogram for the LiFePO_4_/GNPE-1/Li half−cell between −0.5 to 6 V at a rate of 0.1 mV s^−1^.

To calculate the σ values of GNPEs, the EIS values of electrolyte samples were collected using a asymmetric dummy cell, where stainless steel was used as a blocking electrode. Nyquist plots were fitted with a common equivalent electric circuit model in Z-view. The resulting values are displayed in Figure S3. The σ was calculated using the equation σ = *L*/*R_b_ A*, where L was the thickness and *R_b_* and *A* were the bulk resistance and contact area of polymer electrolytes, respectively. Figure 3a displays plots of temperature versus the σ values of the GNPEs. The σ values monotonically increased with increasing temperature of measurement. It was found that the σ values were between 10^−4^ and 10^−5^ S cm^−1^. It is well known that the mobile ability of polymer segments can be enhanced at high temperatures due to increased chain flexibility. As a result, lithium ions could be transported easily in polymer electrolytes at high temperatures [58]. Moreover, the obtained electrolytes of GNPE-1, GNPE-1.5, and GNPE-2 had σ values of 2.63 × 10^−4^ S cm^−1^, 1.17 × 10^−4^ S cm^−1^, and 6.21 × 10^−5^ S cm^−1^ at RT, respectively. Surprisingly, these σ values decreased as the LiFSI concentration increased at low temperatures. GNPE-2 revealed a little higher ionic conductivity than GNPE-1.5 from 50 to 80 °C. However, GNPE-1 still maintained the highest σ values at all measurement temperatures. This might be due to the fact that too much conjugation between FSI^-^ and epoxy can restrict polymer chain movement. The free volume in the polymer matric could also decline, thus affecting ion transport in polymer electrolytes. The highest value of GNPE-1 also conformed to the XRD result that GNPE-1 had the largest FWHM and smallest particle size, suggesting sufficient free volume in the polymer matrix. The *t*_Li_^+^ of GNPEs was calculated from the Bruce–Vincent equation using AC impedance spectroscopy combined with DC polarization at RT (Table S1). These electrolytes were sandwiched between lithium chips using a CR2032 coin cell. Figure 3b–d exhibit the chronoamperometry profiles and AC impedance of GNPEs before and after polarization under a voltage of 10 mV. The resultant AC impedance values for GNPE-1, GNPE-1.5, and GNPE-2 were 0.58, 0.46, and 0.42, respectively. GNPE-1 still presented the best lithium transport behavior, possibly due to its appropriate LiFSI concentration. The comparison of the ionic conductivity and the *t*_Li_^+^ of the published gel polymer electrolyte is demonstrated in Table S2. 

Linear sweep voltammetry (LSV) was conducted to further confirm the maximum irreversible oxidative voltage and electrochemical stable window of GNPEs. This parameter is related to the energy density and capacity of the LIBs [59]. The obtained GNPEs were assembled in a structure of stainless steel (SS)/ GNPEs/ Li foil, wherein SS was used as a working electrode and Li foil was used as both counter and reference electrode. Figure 3e displays the LSV profiles of GNPEs from 0 to 6 V at a sweep rate of 1 mV s^−1^. GNPE-1 exhibited an anodic stability up to 4.15 V vs. Li/Li^+^. GNPE-1.5 showed a lower stable voltage, below 4 V vs. Li/Li^+^. GNPE-2 decomposed at a pretty low voltage, ca. 1.5 V vs. Li/Li^+^. These results indicate that GNPE-1 can meet the voltage requirements for LFP-based lithium-ion batteries (2.5–4.2 V). In addition, cyclic voltammetry (CV) was performed to ascertain the plating and stripping of lithium ions. Figure 3f illustrates the cyclic voltammogram of the LiFePO_4_/GNPE-1/Li foil half-cell conducted at a scan rate of 0.1 mV s^−1^. The voltammograms showed anodic and cathodic peaks at ca. 4.0 and 2.8 V vs. Li/Li^+^, which corresponded to Li-ions delithiation (oxidation) and lithiation (reduction), respectively. Noticeably, these peaks from the 3rd to the 10th cycle remained at similar voltages, indicating the excellent reversibility of redox reactions at the interface of the GNPE/electrodes.

**Figure 4:**
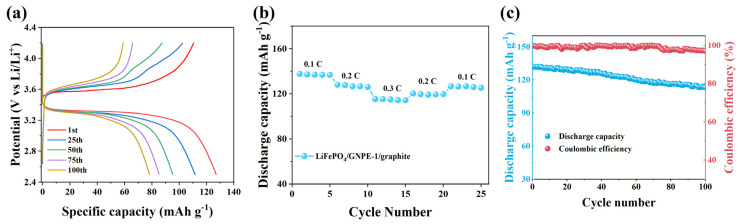
(**a**) Voltage profile of the LFP/ GNPE-1/graphite battery at selected cycles under 0.2 C, (**b**) rate performance of the LFP/GNPE-1/graphite battery, and (**c**) Coulombic efficiency and discharge capacity of the LFP/GNPE-1/graphite battery for 0.2 C after 100 cycles at RT.

The rate and long-cycle performance are the most critical parameters for rechargeable LIBs. To evaluate the cell performances of electrolytes, the charging and discharging of GNPE-1 was carried out from 2.5 to 4.2 V at RT to obtain its best ionic conductivity and electrochemical window. Figure 4a shows the charging and discharging profiles of the LiFePO_4_/GNPE-1/graphite CR 2032 coin cell. GNPE-1 presented the maximum specific discharge capacity of 127.03 mAh g^−1^ at 0.2 C. Its capacity declined with increased cycles, which dropped to 78.28 mAh g^−1^ after 100 cycles (61.62% of the initial capacity). As shown in Figure 4b, the discharge capacity returned to 126.54 mAh g^−1^ when the current density rate was 0.1 C again, suggesting the excellent reversibility and stability of GNPE-1 in rechargeable LIBs. As shown in Figure 4c, the cell with GNPE-1 showed a Coulombic efficiency of over 97% and a capacity of 98 mAh g^−1^ at 0.2 C after 100 cycles, which could maintain 85% of its initial capacity. The cycle performance further confirmed that the application of GNPE-1 to LIBs had a satisfactory stability in a long cycle. The promising initial capacity of the cell with GNPE-1 might be ascribed to the excellent wettability of polymer electrolyte on the LiFePO_4_ electrode, forming a stable interface on the electrode. 

**Figure 5:**
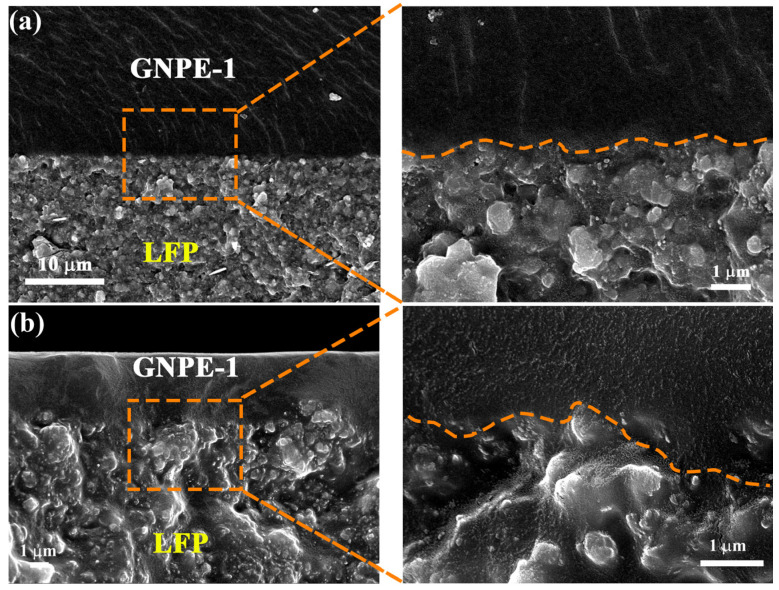
SEM images of a cross-section for GNPE-1 before (**a**) and after (**b**) 100 cycles on the LFP cathode.

The interface between GNPE-1 and the LiFePO_4_ electrode before and after the cycling test was compared by FE-SEM. Figure 5a shows the cross-sectional image, displaying that the prepared GNPE (top) is in close contact with the LFP cathode (bottom), which is attributed to the precursor electrolyte solution penetrating the LFP cathode well. The aging process resulted in an intimate contact interface with the electrode after in situ polymerization. Such connections between the electrode and electrolyte were also observed after the cycle test. Figure 5b displays a cross-sectional SEM image of GNPE-1 after 100 cycles of the cell test. The GNPE-1 layer still presented excellent compatibility with the LPF cathode and maintained its mechanical integrity after the cycle test. These results not only exhibit that the in situ prepared GNPE-1 has excellent mechanical stability, but also hint that the formed crosslinked network can suppress lithium dendrite growth and ensure a long lifespan of LIBs. Additionally, the electrolyte element analysis using EDS outlined that all components were homogeneously distributed on the electrode because all materials in the precursor solution were mixed uniformly (Figure S4).

**Figure 6:**
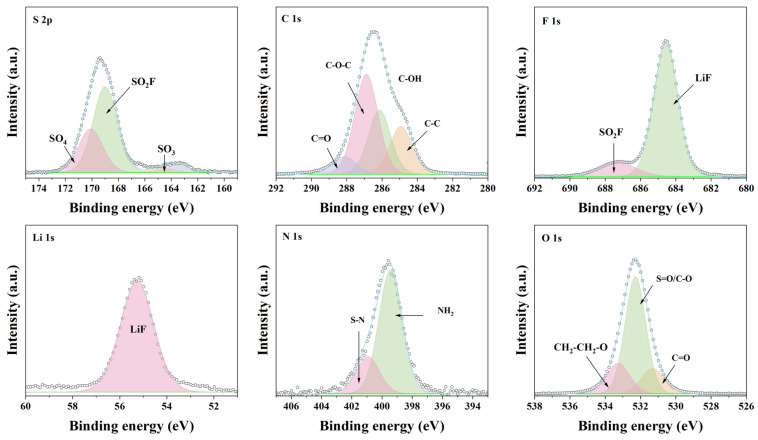
High-resolution XPS characterization (S 2p, C 1s, F 1s, Li 1s, N 1s, and O 1s) of GNPE-1 components on cycled graphite anodes.

Furthermore, XPS was selected for the chemical surface composition investigation of the graphite anode after the cycling test. To the best of our knowledge, LiF was formed after LiFSI electrochemical reduction through S-F bond cleavage [60]. This result can explain the high amount of LiF in the SEI when an imide-based lithium salt is applied to the electrolyte [61]. In Figure 6, the LiF peaks in F 1s (684.5 eV) and Li 1s (55.2 eV) implied that the SEI layer formed on the graphite anode after the cell test [49]. The dominant peak at 687.29 eV in the F 1s spectra was attributed to the SO_2_F of LiFSI, which could also be found at the peak of 168.99 eV in the S 2p spectra [62,63]. Moreover, the S=O species located at 539.29 eV in the O1s spectra overlapped with the C-O species. Turning to the C 1s scan, the peaks from 284.83 to 289.78 eV were mainly assigned to the solvent of the EC/DEC and CH_2_-CH_2_-O chain after epoxy ring-opening polymerization. The corresponding peak of the CH_2_-CH_2_-O chain was also surveyed in the O 1s spectra at 531.59 eV according to a previous report [64,65]. Finally, the N 1s spectra exhibited two peaks at 400.93 and 399.44 eV corresponding to S-N and NH_2_ from the FSI^−^ anion [66].

## 3. Conclusions

In this work, gel network polymer electrolytes, GNPEs, were prepared via a tri-epoxide functional monomer and LiFSI with a little EC and EDC. The polymerization was catalyzed by LiFSI under thermal (60 °C) conditions using an in situ process. As expected, impurity-free gel polymer electrolytes were obtained, showing excellent interface compatibility with the electrodes. Moreover, the prepared GNPEs showed comparable thermal stability (>150 °C) to the liquid electrolyte, having a relatively high σ (2.63 × 10^−4^ S cm^−1^) and *t*_Li_^+^(0.58) at RT. The battery of LiFePO_4_/GNPE-1/graphite demonstrated an excellent initial discharge capacity of 127.03 mAh g^−1^ at 0.2C. It also possessed a good cycling Coulombic efficiency (>97%) and rate performance. The as-prepared GNPE also prevented lithium dendrites from destroying batteries to a certain degree and forming an SEI layer on the anode. In summary, the fundamental findings of the present work could improve our conventional knowledge of epoxy-based gel polymer electrolytes and provide a facial method to prepare high-performance electrolytes for LIBs.

## 4. Materials and Methods

### 4.1. Sources

Trimethylolpropane triglycidyl ether (TMPTE, technical grade) and LiFSI (ultradry, 99.9%) were obtained from Sigma-Aldrich (Seoul, South Korea). Solvents of ethylene carbonate (EC, ≥99%, acid <10 ppm, H_2_O <10 ppm), diethyl carbonate (DEC, ≥99%, acid <10 ppm, H_2_O <10 ppm), and N-methyl-2-pyrrolidone (NMP) were procured from Sigma-Aldrich (Seoul, South Korea). Coin cell cases, aluminum foil, copper foil, and related components were purchased from the MTI Corporation (Richmond, CA, USA). Lithium iron (II) phosphate (LiFePO_4_), carbon black (super P, Timcal), and poly(vinylidene fluoride) (PVDF, power) were purchased from Alfa Aesar (Seoul, South Korea).

### 4.2. Electrodes Preparation

LiFePO_4_, carbon black, and PVDF were mixed at a mass ratio of 80:10:10. NMP was added as a solvent. A slurry was obtained after thoroughly grinding with a ball mill. The obtained slurry was used to coat aluminum foil followed by drying at 120 °C to remove the solvent of NMP. Active materials were loaded on the foil at 12 mg cm^−2^. The obtained cathode foil was punched into circular pieces with a diameter of 16 mm followed by drying at 100 °C for 12 h before cell assembly. The graphite anode was prepared with a similar process. The slurry contained graphite, carbon black, and PVDF at a mass ratio of 85:5:10.

### 4.3. In Situ Polymerization of GNPEs and Cell Assembly

To prepare the precursor electrolyte solution, various mole ratios of LiFSI were dissolved in TMPTE and EC/DEC (50:50/*v*:*v*) mixture. The precursor solution was then dripped on the LFP cathode and polymerized at 60 °C for 12 h. The obtained in situ electrolytes were named GNPE-1, GNPE-1.5, and GNPE-2, respectively. The numerical suffix indicates the molar concentration of LiFSI. The LiFePO_4_/GNPE/graphite cell was assembled with a GNPEs-LFP plate. It was then aged at RT for 24 h before measurement. All preparations, including in situ polymerization and cell assembly, were carried out in an argon glove box (H_2_O and O_2_ < 1 ppm). Scheme S1 presents the details of the preparation process.

### 4.4. Instrumentations and Measurements

A Nicolet iS5 infrared Fourier transform (FT IR) spectrometer (Thermo Fisher Scientific, Waltham, MA, USA) was used to monitor the polymerization of the electrolyte in a scan range of 4000 to 500 cm^–1^ with a spectral resolution of 4 cm^–1^. Thermogravimetric analysis (TGA) was performed to evaluate the thermal stability of electrolytes on a TGA-8000 (Perkin Elmer Co., Ltd., Waltham, MA, USA) over a temperature range of RT–500 °C at a heating rate of 10 °C min^−1^ under a nitrogen (N_2_) atmosphere. Differential scanning calorimetry (DSC) for confirming the glass-transition temperature (*T*_g_) was performed with a Perkin-Elmer DSC 6000 (Waltham, MA, USA) at temperatures between −60 and 150 °C with a heating/cooling rate of 10 °C min^−1^ under a N_2_ atmosphere. Interfacial morphology and elemental compositions were investigated with a field emission scanning electron microscope (FE-SEM) and an energy-dispersive X-ray spectroscope (EDS) using a JSM-6700F (JEOL, Tokyo, Japan) at an accelerating voltage of 15.0 kV. Crystal phase and X-ray diffraction (XRD) analyses were performed with a D2 Phaser (Bruker, Leipzig, Germany) with Cu K*α* radiation in a 2*θ* range from 10 to 80° at a speed rate of 2° min^−1^. X-ray photoelectron spectroscopy (XPS) was performed with a K Alpha+ analyzer (Thermo Scientific, Oxford, UK) using monochromatic Al Kα X-rays (hν = 1486.6 eV) with a beam spot size of 400 μm. The wide survey and narrow scan pass energies were 200 and 50 eV, respectively. Data were analyzed on a Ca XPS software (inCAx-sight7421) fitting with a Shirley-type background and calibrated to adventitious C 1s bending energy at 284.6 eV. For electrode sample preparation, the LPF cathode and graphite anode were collected from cells after 100 cycles and cut in the liquid nitrogen to avoid cross-side deformation. Electrochemical impedance spectroscopy (EIS) was performed with an IM6ex, Zahner-Elektrik GmbH & Co. K.G. instrument (Kronach, Germany) between RT and 80 °C at a frequency range of 0.1 Hz to 1 MHz with an alternating current (AC) amplitude of 10 mV. EIS spectra fitting was run on Z-view (version 3.1, Scribner Associates Inc., Southern Pines, NC, USA). At RT, the electrochemical stability window was confirmed by cycle voltammetry (CV) and linear sweep voltammetry (LSV). Data were collected at a voltage of 0–6 V with a scan rate of 1 and 0.1 mV s^−1^. Cell performance tests were all carried out on an Ivium-n-Stat (Ivium Technologies B.V., Eindhoven, The Netherlands) at RT. The charging/discharging test was performed with a CR2032 coin cell at cutoff voltages of 2.5−4.2 V vs. Li/Li^+^.

## Data Availability

The data presented in this study are openly available in article.

## Supplementary Materials

The following supporting information can be downloaded at: https://www.mdpi.com/article/10.3390/gels10010040/s1, Figure S1: GNPEs preparation and cell assembly structure; Figure S2: Chemical structure of TMPTE and FT IR resulting spectra of electrolytes with different times; Figure S3: Nyquist plots of electrolytes at various temperatures and an equivalent electric circuit model; Figure S4: Elemental mapping and EDS analysis of GNPE-1; Table S1: Currents and resistances of the electrolytes for t*_Li_^+^* calculation; Table S2: Comparison of electrochemical properties of polymer electrolyte from reported works [67,68,69,70,71,72].

## References

[1] von Bülow F., Meisen T. (2023). A Review on Methods for State of Health Forecasting of Lithium-Ion Batteries Applicable in Real-World Operational Conditions. J. Energy Storage.

[2] Zhu A., Bian X., Han W., Cao D., Wen Y., Zhu K., Wang S. (2023). The Application of Deep Eutectic Solvents in Lithium-Ion Battery Recycling: A Comprehensive Review. Resour. Conserv. Recycl..

[3] Tang S., Guo W., Fu Y. (2021). Advances in Composite Polymer Electrolytes for Lithium Batteries and Beyond. Adv. Energy Mater..

[4] Lv C., Zhou X., Zhong L., Yan C., Srinivasan M., Seh Z.W., Liu C., Pan H., Li S., Wen Y. (2022). Machine Learning: An Advanced Platform for Materials Development and State Prediction in Lithium-Ion Batteries. Adv. Mater..

[5] Edge J.S., O’Kane S., Prosser R., Kirkaldy N.D., Patel A.N., Hales A., Ghosh A., Ai W., Chen J., Yang J. (2021). Lithium Ion Battery Degradation: What You Need to Know. Phys. Chem. Chem. Phys..

[6] Zhang Y.S., Courtier N.E., Zhang Z., Liu K., Bailey J.J., Boyce A.M., Richardson G., Shearing P.R., Kendrick E., Brett D.J.L. (2022). A Review of Lithium-Ion Battery Electrode Drying: Mechanisms and Metrology. Adv. Energy Mater..

[7] Baum Z.J., Bird R.E., Yu X., Ma J. (2022). Lithium-Ion Battery Recycling—Overview of Techniques and Trends. ACS Energy Lett..

[8] Cui Z., Wang L., Li Q., Wang K. (2022). A Comprehensive Review on the State of Charge Estimation for Lithium-Ion Battery Based on Neural Network. Int. J. Energy Res..

[9] Mao S., Wu Q., Ma F., Zhao Y., Wu T., Lu Y. (2021). Advanced Liquid Electrolytes Enable Practical Applications of High-Voltage Lithium-Metal Full Batteries. Chem. Commun..

[10] Feng X., Ouyang M., Liu X., Lu L., Xia Y., He X. (2018). Thermal Runaway Mechanism of Lithium Ion Battery for Electric Vehicles: A Review. Energy Storage Mater..

[11] Wang Q., Ping P., Zhao X., Chu G., Sun J., Chen C. (2012). Thermal Runaway Caused Fire and Explosion of Lithium Ion Battery. J. Power Sources.

[12] Liu K., Liu Y., Lin D., Pei A., Cui Y. (2018). Materials for Lithium-Ion Battery Safety. Sci. Adv..

[13] Li Q., Chen J., Fan L., Kong X., Lu Y. (2016). Progress in Electrolytes for Rechargeable Li-Based Batteries and Beyond. Green Energy Environ..

[14] Kim J.Y., Shin D.O., Chang T., Kim K.M., Jeong J., Park J., Lee Y.M., Cho K.Y., Phatak C., Hong S. (2019). Effect of the Dielectric Constant of a Liquid Electrolyte on Lithium Metal Anodes. Electrochim. Acta.

[15] Xu K. (2014). Electrolytes and Interphases in Li-Ion Batteries and Beyond. Chem. Rev..

[16] Zhao Q., Zhang Y., Tang F., Zhao J., Li S. (2017). Mixed Salts of Lithium Difluoro (Oxalate) Borate and Lithium Tetrafluorobotate Electrolyte on Low-Temperature Performance for Lithium-Ion Batteries. J. Electrochem. Soc..

[17] Zhao W., Ren F., Yan Q., Liu H., Yang Y. (2020). A Facile Synthesis of Non-Aqueous LiPO2F2 Solution as the Electrolyte Additive for High Performance Lithium Ion Batteries. Chin. Chem. Lett..

[18] Giffin G.A. (2016). Ionic Liquid-Based Electrolytes for “beyond Lithium” Battery Technologies. J. Mater. Chem. A Mater..

[19] Wang Y., Zhong W.H. (2015). Development of Electrolytes towards Achieving Safe and High-Performance Energy-Storage Devices: A Review. ChemElectroChem.

[20] Xi G., Xiao M., Wang S., Han D., Li Y., Meng Y. (2021). Polymer-Based Solid Electrolytes: Material Selection, Design, and Application. Adv. Funct. Mater..

[21] Deng K., Zeng Q., Wang D., Liu Z., Qiu Z., Zhang Y., Xiao M., Meng Y. (2020). Single-Ion Conducting Gel Polymer Electrolytes: Design, Preparation and Application. J. Mater. Chem. A Mater..

[22] Jiang Y., Yan X., Ma Z., Mei P., Xiao W., You Q., Zhang Y. (2018). Development of the PEO Based Solid Polymer Electrolytes for All-Solid State Lithium Ion Batteries. Polymers.

[23] Wang C., Yang Y., Liu X., Zhong H., Xu H., Xu Z., Shao H., Ding F. (2017). Suppression of Lithium Dendrite Formation by Using LAGP-PEO (LiTFSI) Composite Solid Electrolyte and Lithium Metal Anode Modified by PEO (LiTFSI) in All-Solid-State Lithium Batteries. ACS Appl. Mater. Interfaces.

[24] Zheng Y., Yao Y., Ou J., Li M., Luo D., Dou H., Li Z., Amine K., Yu A., Chen Z. (2020). A Review of Composite Solid-State Electrolytes for Lithium Batteries: Fundamentals, Key Materials and Advanced Structures. Chem. Soc. Rev..

[25] An Y., Han X., Liu Y., Azhar A., Na J., Nanjundan A.K., Wang S., Yu J., Yamauchi Y. (2022). Progress in Solid Polymer Electrolytes for Lithium-Ion Batteries and Beyond. Small.

[26] Zhou D., Shanmukaraj D., Tkacheva A., Armand M., Wang G. (2019). Polymer Electrolytes for Lithium-Based Batteries: Advances and Prospects. Chem.

[27] Hikima K., Huy Phuc N.H., Tsukasaki H., Mori S., Muto H., Matsuda A. (2020). High Ionic Conductivity of Multivalent Cation Doped Li6PS5Cl Solid Electrolytes Synthesized by Mechanical Milling. RSC Adv..

[28] Yu C., van Eijck L., Ganapathy S., Wagemaker M. (2016). Synthesis, Structure and Electrochemical Performance of the Argyrodite Li6PS5Cl Solid Electrolyte for Li-Ion Solid State Batteries. Electrochim. Acta.

[29] Park K.H., Bai Q., Kim D.H., Oh D.Y., Zhu Y., Mo Y., Jung Y.S. (2018). Design Strategies, Practical Considerations, and New Solution Processes of Sulfide Solid Electrolytes for All-Solid-State Batteries. Adv. Energy Mater..

[30] Ma C., Cui W., Liu X., Ding Y., Wang Y. (2022). In Situ Preparation of Gel Polymer Electrolyte for Lithium Batteries: Progress and Perspectives. InfoMat.

[31] Bocharova V., Sokolov A.P. (2020). Perspectives for Polymer Electrolytes: A View from Fundamentals of Ionic Conductivity. Macromolecules.

[32] Wang W., Li Z., Huang H., Li W., Wang J. (2022). Facile Design of Novel Nanocellulose-Based Gel Polymer Electrolyte for Lithium-Ion Batteries Application. Chem. Eng. J..

[33] Wei J., Yue H., Shi Z., Li Z., Li X., Yin Y., Yang S. (2021). In Situ Gel Polymer Electrolyte with Inhibited Lithium Dendrite Growth and Enhanced Interfacial Stability for Lithium-Metal Batteries. ACS Appl. Mater. Interfaces.

[34] Lv P., Yang J., Liu G., Liu H., Li S., Tang C., Mei J., Li Y., Hui D. (2017). Flexible Solid Electrolyte Based on UV Cured Polyurethane Acrylate/Succinonitrile-Lithium Salt Composite Compatibilized by Tetrahydrofuran. Compos. B Eng..

[35] Oh B., Jung W.I., Kim D.W., Rhee H.W. (2002). Preparation of UV Curable Gel Polymer Electrolytes and Their Electrochemical Properties. Bull. Korean Chem. Soc..

[36] Mindemark J., Lacey M.J., Bowden T., Brandell D. (2018). Beyond PEO—Alternative Host Materials for Li+-Conducting Solid Polymer Electrolytes. Prog. Polym. Sci..

[37] Xue Z., He D., Xie X. (2015). Poly(Ethylene Oxide)-Based Electrolytes for Lithium-Ion Batteries. J. Mater. Chem. A Mater..

[38] Cheng H., Zhu J., Jin H., Gao C., Liu H., Cai N., Liu Y., Zhang P., Wang M. (2020). In Situ Initiator-Free Gelation of Highly Concentrated LiFSI-DOL Solid Polymer Electrolyte for High Performance Lithium-Metal Batteries. Mater. Today Energy.

[39] Foran G., Verdier N., Lepage D., Prébé A., Aymé-Perrot D., Dollé M. (2021). Thermal and Electrochemical Properties of Solid Polymer Electrolytes Prepared via Lithium Salt-Catalyzed Epoxide Ring Opening Polymerization. Appl. Sci..

[40] Zhi M., Liu Q., Chen H., Chen X., Feng S., He Y. (2019). Thermal Stability and Flame Retardancy Properties of Epoxy Resin Modified with Functionalized Graphene Oxide Containing Phosphorus and Silicon Elements. ACS Omega.

[41] Xue C., Qin Y., Fu H., Fan J. (2022). Thermal Stability, Mechanical Properties and Ceramization Mechanism of Epoxy Resin/Kaolin/Quartz Fiber Ceramifiable Composites. Polymers.

[42] Yeasmin F., Mallik A.K., Chisty A.H., Robel F.N., Shahruzzaman M., Haque P., Rahman M.M., Hano N., Takafuji M., Ihara H. (2021). Remarkable Enhancement of Thermal Stability of Epoxy Resin through the Incorporation of Mesoporous Silica Micro-Filler. Heliyon.

[43] Herzberger J., Niederer K., Pohlit H., Seiwert J., Worm M., Wurm F.R., Frey H. (2016). Polymerization of Ethylene Oxide, Propylene Oxide, and Other Alkylene Oxides: Synthesis, Novel Polymer Architectures, and Bioconjugation. Chem. Rev..

[44] Crivello J.V., Lee J.L. (1985). Recent Advances in Thermally and Photochemically Initiated Cationic Polymerization. Polym. J..

[45] Crivello J.V. (2009). Radical-Promoted Visible Light Photoinitiated Cationic Polymerization of Epoxides. J. Macromol. Sci. Part A Pure Appl. Chem..

[46] Stanford J.L., Ryan A.J., Yang Y. (2001). Photoinitiated Cationic Polymerization of Epoxides. Polym. Int..

[47] Zhou D., Fan L.Z., Fan H., Shi Q. (2013). Electrochemical Performance of Trimethylolpropane Trimethylacrylate-Based Gel Polymer Electrolyte Prepared by in Situ Thermal Polymerization. Electrochim. Acta.

[48] Wang Q.J., Zhang P., Wang B., Fan L.Z. (2021). A Novel Gel Polymer Electrolyte Based on Trimethylolpropane Trimethylacrylate/Ionic Liquid via in Situ Thermal Polymerization for Lithium-Ion Batteries. Electrochim. Acta.

[49] Kang S.J., Park K., Park S.H., Lee H. (2018). Unraveling the Role of LiFSI Electrolyte in the Superior Performance of Graphite Anodes for Li-Ion Batteries. Electrochim. Acta.

[50] Lu H., Zeng S., Zhao D., Wang J., Quan Y., Xu F., Li F., Li S. (2021). Optimizing the Composition of LiFSI-Based Electrolytes by a Method Combining Simplex with Normalization. RSC Adv..

[51] Huangzhang E., Zeng X., Yang T., Liu H., Sun C., Fan Y., Hu H., Zhao X., Zuo X., Nan J. (2022). A Localized High-Concentration Electrolyte with Lithium Bis(Fluorosulfonyl) Imide (LiFSI) Salt and F-Containing Cosolvents to Enhance the Performance of Li||LiNi_0.8_Co_0.1_Mn_0.1_O_2_ Lithium Metal Batteries. Chem. Eng. J..

[52] Chen X., Yao N., Zeng B.S., Zhang Q. (2021). Ion–Solvent Chemistry in Lithium Battery Electrolytes: From Mono-Solvent to Multi-Solvent Complexes. Fundam. Res..

[53] Wang A.A., Greenbank S., Li G., Howey D.A., Monroe C.W. (2022). Current-Driven Solvent Segregation in Lithium-Ion Electrolytes. Cell Rep. Phys. Sci..

[54] Zeng Z., Liang W.-I., Liao H.-G., Xin H.L., Chu Y.-H., Zheng H. (2014). Visualization of Electrode–Electrolyte Interfaces in LiPF6/EC/DEC Electrolyte for Lithium Ion Batteries via in Situ TEM. Nano Lett..

[55] Sampson K., Paik A., Duvall B., Whalen D.L. (2004). Transition-State Effects in Acid-Catalyzed Aryl Epoxide Hydrolyses. J. Org. Chem..

[56] Bulut U., Crivello J.V. (2005). Investigation of the Reactivity of Epoxide Monomers in Photoinitiated Cationic Polymerization. J. Polym. Sci. A Polym. Chem..

[57] Shi F., Ross P.N., Somorjai G.A., Komvopoulos K. (2017). The Chemistry of Electrolyte Reduction on Silicon Electrodes Revealed by in Situ ATR-FTIR Spectroscopy. J. Phys. Chem. C.

[58] Yi L., Zou C., Chen X., Liu J., Cao S., Tao X., Zang Z., Liu L., Chang B., Shen Y. (2022). One-Step Synthesis of PVDF-HFP/PMMA-ZrO2Gel Polymer Electrolyte to Boost the Performance of a Lithium Metal Battery. ACS Appl. Energy Mater..

[59] Arya A., Sharma A.L. (2020). A Glimpse on All-Solid-State Li-Ion Battery (ASSLIB) Performance Based on Novel Solid Polymer Electrolytes: A Topical Review. J. Mater. Sci..

[60] Philippe B., Dedryvère R., Gorgoi M., Rensmo H., Gonbeau D., Edström K. (2013). Improved Performances of Nanosilicon Electrodes Using the Salt LiFSI: A Photoelectron Spectroscopy Study. J. Am. Chem. Soc..

[61] Eshetu G.G., Judez X., Li C., Martinez-Ibañez M., Gracia I., Bondarchuk O., Carrasco J., Rodriguez-Martinez L.M., Zhang H., Armand M. (2018). Ultrahigh Performance All Solid-State Lithium Sulfur Batteries: Salt Anion’s Chemistry-Induced Anomalous Synergistic Effect. J. Am. Chem. Soc..

[62] Sharova V., Moretti A., Diemant T., Varzi A., Behm R.J., Passerini S. (2018). Comparative Study of Imide-Based Li Salts as Electrolyte Additives for Li-Ion Batteries. J. Power Sources.

[63] Siva G., Aziz M.A., Kumar G.G. (2018). Engineered Tubular Nanocomposite Electrocatalysts Based on CuS for High-Performance, Durable Glucose Fuel Cells and Their Stack. ACS Sustain. Chem. Eng..

[64] Jiao S., Ren X., Cao R., Engelhard M.H., Liu Y., Hu D., Mei D., Zheng J., Zhao W., Li Q. (2018). Stable Cycling of High-Voltage Lithium Metal Batteries in Ether Electrolytes. Nat. Energy.

[65] Kumar G.G., Chung S.H., Kumar T.R., Manthiram A. (2018). Three-Dimensional Graphene–Carbon Nanotube–Ni Hierarchical Architecture as a Polysulfide Trap for Lithium–Sulfur Batteries. ACS Appl. Mater. Interfaces.

[66] Budi A., Basile A., Opletal G., Hollenkamp A.F., Best A.S., Rees R.J., Bhatt A.I., O’Mullane A.P., Russo S.P. (2012). Study of the Initial Stage of Solid Electrolyte Interphase Formation upon Chemical Reaction of Lithium Metal and N -Methyl- N -Propyl-Pyrrolidinium-Bis(Fluorosulfonyl)Imide. J. Phys. Chem. C.

[67] Yao W., Zhang Q., Qi F., Zhang J., Liu K., Li J., Chen W., Du Y., Jin Y., Liang Y. (2019). Epoxy Containing Solid Polymer Electrolyte for Lithium Ion Battery. Electrochim. Acta.

[68] Ma Y., Sun Q., Wang S., Zhou Y., Song D., Zhang H., Shi X., Zhang L. (2022). Li Salt Initiated In-Situ Polymerized Solid Polymer Electrolyte: New Insights via in-Situ Electrochemical Impedance Spectroscopy. Chem. Eng. J..

[69] Jin L., Jang G., Lim H., Zhang W., Kim W., Jang H. (2022). An In Situ Polymeric Electrolyte with Low Interfacial Resistance on Electrodes for Lithium-Ion Batteries. Adv. Mater. Interfaces.

[70] Yu T.Y., Yeh S.C., Lee J.Y., Wu N.L., Jeng R.J. (2021). Epoxy-Based Interlocking Membranes for All Solid-State Lithium Ion Batteries: The Effects of Amine Curing Agents on Electrochemical Properties. Polymers.

[71] Li M., Li H., Lan J.L., Yu Y., Du Z., Yang X. (2018). Integrative Preparation of Mesoporous Epoxy Resin-Ceramic Composite Electrolytes with Multilayer Structure for Dendrite-Free Lithium Metal Batteries. J. Mater. Chem. A Mater..

[72] Li S., Jiang H., Tang T., Nie Y., Zhang Z., Zhou Q. (2018). Improved Electrochemical and Mechanical Performance of Epoxy-Based Electrolytes Doped with Mesoporous TiO_2_. Mater. Chem. Phys..

