# Supplementary material for: Crosslinked Gel Polymer Electrolyte from Trimethylolpropane Triglycidyl Ether by In Situ Polymerization for Lithium-Ion Batteries
Source: Gels. 2024 Jan 2;10(1):40. doi: 10.3390/gels10010040 (PMC10815923; doi:10.3390/gels10010040)
Supplement: Supplementary file 1 [file gels-10-00040-s001.zip › gels-2770127-supplementary.pdf]

Crosslinked Gel Polymer Electrolyte from Trimethylolpropane Triglycidyl Ether by In Situ Polymerization for Lithium-Ion Batteries

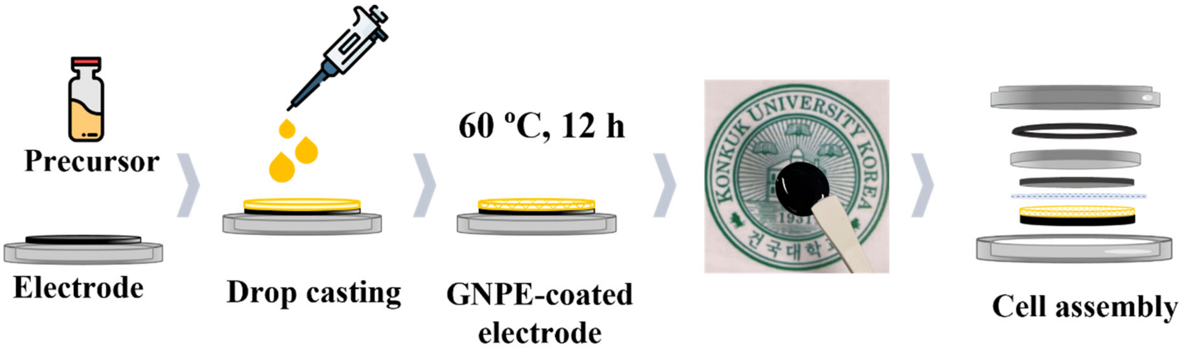

Figure S1. GNPEs preparation and cell assembly structure.

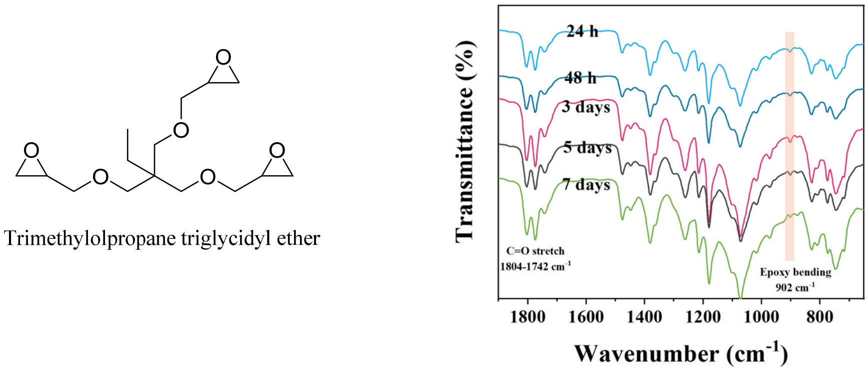

Figure S2. Chemical structure of TMPTE and FT-IR resulting spectra of electrolyte with different times.

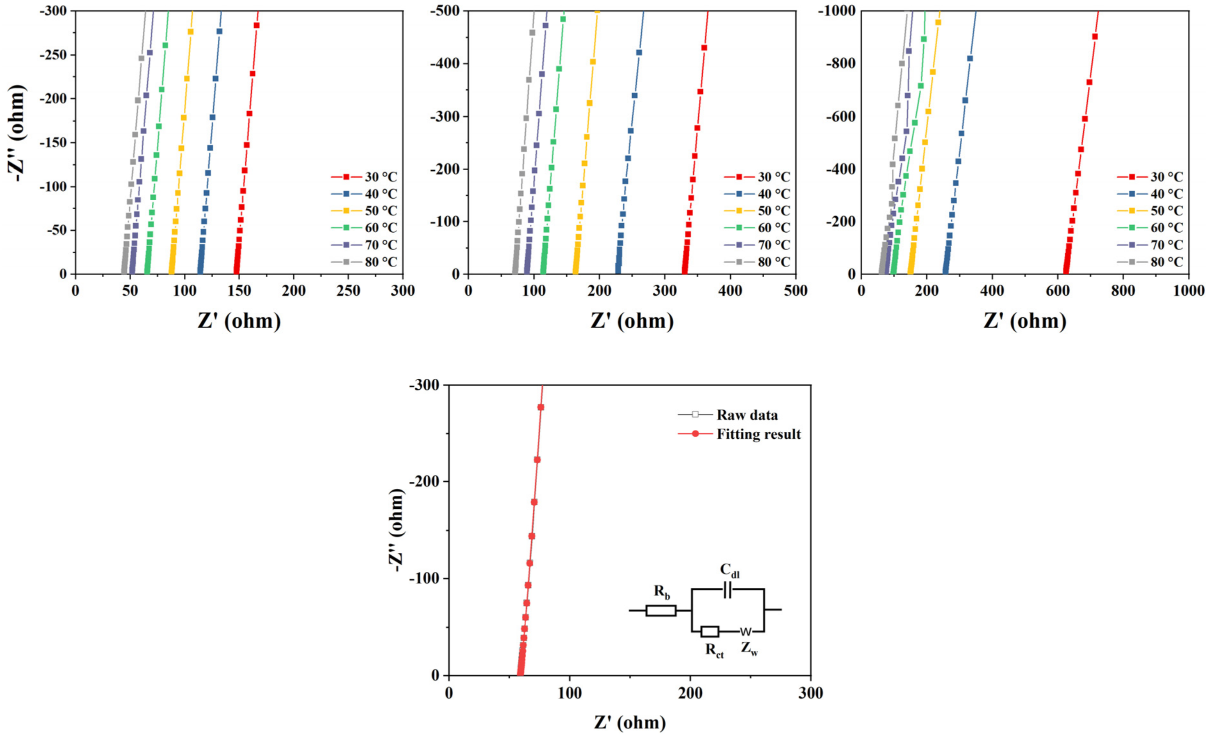

Figure S3. Nyquist plots of electrolytes at various temperatures and equivalent electric circuit model.

Table S1. Currents and resistances of the electrolytes for t_{Li}^+ calculation.

Electrolyte	Is (A)	I_0(A)	ΔV(mV)	R_0(ohm)	Rs(ohm)	t_{Li}^+
GNPE-1	2.18E-06	3.71E-06	0.1	145	213	0.58
GNPE-1.5	9.80E-07	2.11E-06	0.1	320	532	0.46
GNPE-2	1.03E-06	2.39E-06	0.1	640	897	0.42

Bruce–Vincent–Evans Equation:

$$t_{Li}^+ = I_s\,(\Delta V - I_0R_0)/\,I_0\,(\Delta V - I_sR_s)$$

where I_0 and I_s are the initial and steady-state current, and R_0 and R_s are denoted as the interfacial resistance between electrode and electrolyte of the symmetrical cell before and after polarization, respectively.

Table S2. Comparison of electrochemical properties of polymer electrolyte from reported works.

Polymer Electrolyte	Molecular Structure	σ S cm^{-1}	t^+	Ref
**SPE**	PGO	2.56×10^{-8}	0.549	[1]
	HPGO	4.07×10^{-7}	0.693	
**In situ gel polymer**	PGTE	4.16×10^{-4}	0.122	[2]
**In situ polymer**	PSEPE	1.16×10^{-4}	0.61	[3]
**SPE**	PGA	5.61×10^{-4}	0.43	[4]
**GPE**	DGEBA/L LTO	2.02×10^{-3}	0.82	[5]
**GPE**	TiO_2/Epoxy-based composite	1.1×10^{-4}	0.661	[6]
**In situ gel polymer**	GNPE-1	2.63×10^{-4}	0.58	This work
	GNPE-1.5	1.17×10^{-4}	0.46	
	GNPE-2	6.21×10^{-5}	0.42	

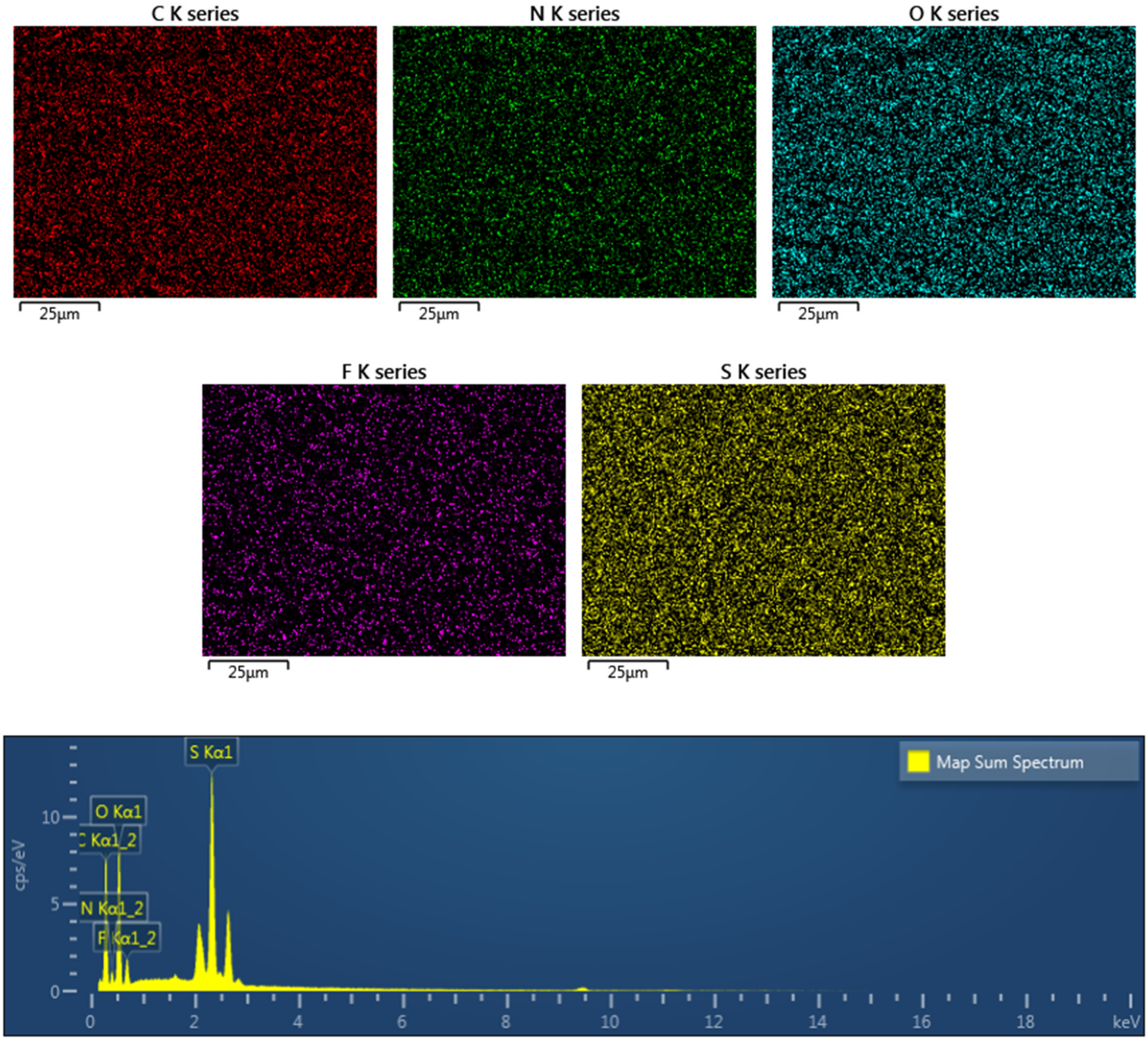

Element	Wt.%	Wt.% Sigma
C	42.71	0.54
N	11.3	0.7
O	30.37	0.43
F	5.37	0.21
S	10.24	0.14
Total:	100	

Figure S4. Elemental mapping and EDS analysis of GNPE-1.

References

1. Yao, W.; Zhang, Q.; Qi, F.; Zhang, J.; Liu, K.; Li, J.; Chen, W.; Du, Y.; Jin, Y.; Liang, Y.; et al. Epoxy Containing Solid Polymer Electrolyte for Lithium Ion Battery. *Electrochim Acta* **2019**, *318*, 302–313, doi:10.1016/j.electacta.2019.06.069.

2. Ma, Y.; Sun, Q.; Wang, S.; ZPhou, Y.; Song, D.; Zhang, H.; Shi, X.; Zhang, L. Li Salt Initiated In-Situ Polymerized Solid Polymer Electrolyte: New Insights via in-Situ Electrochemical Impedance Spectroscopy. *Chemical Engineering Journal* **2022**, *429*, 132483, doi:10.1016/j.cej.2021.132483.

3. Jin, L.; Jang, G.; Lim, H.; Zhang, W.; Kim, W.; Jang, H. An In Situ Polymeric Electrolyte with Low Interfacial Resistance on Electrodes for Lithium-Ion Batteries. *Adv Mater Interfaces* **2022**, *9*, 1–11, doi:10.1002/admi.202101958.

4. Yu, T.Y.; Yeh, S.C.; Lee, J.Y.; Wu, N.L.; Jeng, R.J. Epoxy-Based Interlocking Membranes for All Solid-State Lithium Ion Batteries: The Effects of Amine Curing Agents on Electrochemical Properties. *Polymers (Basel)* **2021**, *13*, doi:10.3390/polym13193244.

5. Li, M.; Li, H.; Lan, J. Le; Yu, Y.; Du, Z.; Yang, X. Integrative Preparation of Mesoporous Epoxy Resin-Ceramic Composite Electrolytes with Multilayer Structure for Dendrite-Free Lithium Metal Batteries. *J Mater Chem A Mater* **2018**, *6*, 19094–19106, doi:10.1039/c8ta06341a.

6. Li, S.; Jiang, H.; Tang, T.; Nie, Y.; Zhang, Z.; Zhou, Q. Improved Electrochemical and Mechanical Performance of Epoxy-Based Electrolytes Doped with Mesoporous TiO2. *Mater Chem Phys* **2018**, *205*, 23–28, doi:10.1016/j.matchemphys.2017.10.075.
